# Microscale *In Vitro* Assays for the Investigation of Neutral Red Retention and Ethoxyresorufin-*O*-Deethylase of Biofuels and Fossil Fuels

**DOI:** 10.1371/journal.pone.0163862

**Published:** 2016-09-29

**Authors:** Sebastian Heger, Kerstin Bluhm, Julia Brendt, Philipp Mayer, Nico Anders, Andreas Schäffer, Thomas-Benjamin Seiler, Henner Hollert

**Affiliations:** 1 RWTH Aachen University, Institute for Environmental Research, Department of Ecosystem Analysis, Aachen, Germany; 2 Technical University of Denmark, Department of Environmental Engineering, Kongens Lyngby, Denmark; 3 RWTH Aachen University, Chemical Engineering, Enzyme Process Technology, Aachen, Germany; 4 RWTH Aachen University, Institute for Environmental Research, Chair of Environmental Biology and Chemodynamcis, Aachen, Germany; 5 Chongqing University, College of Resources and Environmental Science, Chongqing, China; 6 Nanjing University, State Key Laboratory of Pollution Control and Resource Reuse, School of the Environment, Nanjing, China; 7 Tongji University, College of Environmental Science and Engineering and State Key Laboratory of Pollution Control and Resource Reuse, Shanghai, China; University of Siena, ITALY

## Abstract

Only few information on the potential toxic effectiveness of biofuels are available. Due to increasing worldwide demand for energy and fuels during the past decades, biofuels are considered as a promising alternative for fossil fuels in the transport sector. Hence, more information on their hazard potentials are required to understand the toxicological impact of biofuels on the environment. In the German Cluster of Excellence “Tailor-made Fuels from Biomass” design processes for economical, sustainable and environmentally friendly biofuels are investigated. In an unique and interdisciplinary approach, ecotoxicological methods are applied to gain information on potential adverse environmental effects of biofuels at an early phase of their development. In the present study, three potential biofuels, ethyl levulinate, 2-methyltetrahydrofuran and 2-methylfuran were tested. Furthermore, we investigated a fossil gasoline fuel, a fossil diesel fuel and an established biodiesel. Two *in vitro* bioassays, one for assessing cytotoxicity and one for aryl hydrocarbon receptor agonism, so called dioxin-like activity, as measured by Ethoxyresorufin-*O*-Deethylase, were applied using the permanent fish liver cell line RTL-W1 (*Oncorhynchus mykiss*). The special properties of these fuel samples required modifications of the test design. Points that had to be addressed were high substance volatility, material compatibility and low solubility. For testing of gasoline, diesel and biodiesel, water accommodated fractions and a passive dosing approach were tested to address the high hydrophobicity and low solubility of these complex mixtures. Further work has to focus on an improvement of the chemical analyses of the fuel samples to allow a better comparison of any effects of fossil fuels and biofuels.

## Introduction

During the last decades, fossil fuels, such as gasoline and diesel, were the main energy source in the transport sector worldwide.[1] In the European Union (EU) the transport sector contributes to 32% of the total energy consumption and is dominated by fossil fuels.[2] This share is predicted to rise further.[3] Beside economic and political considerations, fossil fuels and their combustion products are hazardous for the environment as well as human health, e.g., due to their mutagenic, genotoxic and cancerogenic potential.[4–11] Moreover, the adverse effects of fossil fuel emissions are more and more recognized to impact the global climate.[12]

To reduce the dependency on fossil fuels, they are increasingly replaced by or supplemented with renewable energy carriers, such as biomass-derived fuels, so called biofuels. The introduction of new biofuels is promoted by governmental support, e.g., the European Union, which established a target of at least 10% for energy from renewable sources of the total energy consumption by 2020 (2009/28/EC).[13] The resulting increase in the development and use of biofuels is also under critical discussion. Important topics under consideration are the energy yield of biomass-derived fuels, cost efficiency, competition with food production, greenhouse gas emissions, impact on water resources and land use changes.[14–25]

The ecotoxicological aspects of biofuels, however, are often neglected. Biofuel regulation is so far confined to criteria such as sustainability, land use and CO_2_-emissions, whereas the toxicity of biomass-derived fuels is not explicitly considered.[13] Therefore, purpose-designed fuel molecules will be introduced in the existing infrastructure in significant amounts without previous insight into their potential hazardous effects. However, ecotoxicological *in vitro* bioassays could be applied for rapid and inexpensive screening of certain environmental aspects of biofuels. In a literature review conducted by Bluhm et al.,[26] a considerable lack of data on the ecotoxicological hazard potential of biofuels was elucidated, and ecotoxicological studies were recommended to accompany the development of biofuels following the concept of ‘Green Toxicology’. This approach postulates an economic value in accompanying ecotoxicological assessment for (chemical) product development in the way that early decisions against pursuing further development of a given potential product can save financial resources.[27] It represents a novel approach compared to previous chemical design processes that are often limited to Life-Cycle Analysis (LCA) or mathematical screening tools for assessing persistence or spatial range.[28,29]

Bioassays detect adverse effects of single compounds and complex chemical mixtures on a variety of test organisms, including animals, plants, fungi and bacteria. They allow evaluating effects of complex samples even though none or only few compounds are known or present at very low concentrations. With regard to their limit of detection and their sensitivity, bioassays, such as the 7-ethoxyresorufin-*O*-deethylase (EROD) assay, can even compete with GC-MS analytical procedures.[30,31] Thus, they are ideally suited for the investigation of newly developed biofuel candidates as well as complex fossil fuels.[31] These complex fuel mixtures, however, require special preparation before they can be tested. So called water-accommodated fractions (WAFs) can be used for an investigation of fossil fuels. WAFs contain the soluble part of the investigated fuel sample, which accounts for the majority of toxic effects caused by fossil fuels in aquatic systems[32] and are therefore a commonly applied approach for investigation of fuel samples.[4,33] Unlike water soluble fractions (WSFs),[34–36] no further physical handling, such as filtration or centrifugation of the samples is conducted to avoid uncontrolled chemical loss or change in the sample composition. Additionally, a passive dosing approach can be applied for improved exposure control of hydrophobic organics and their mixtures. A biocompatible polymer is here loaded with the test substance and then used as a partitioning donor for controlling exposure concentrations in the test systems even for low solubility and volatile constituents of the gasoline and diesel fuels. Their continuous resupply into the test medium enables a constant exposure of the test organism throughout the entire test cycle.[37] However, since the value of information obtained by a single bioassay is limited, the application of different bioassays,[38] sometimes in combination with chemical analysis, is often performed.[39,40] In a previous study, ecotoxicological *in vitro* bioassays were identified as suitable tools for an ecotoxicological investigation of biofuel fermentation samples,[41] but further modifications are required for the testing of biofuel candidate substances.

Within the interdisciplinary German Cluster of Excellence “Tailor-made fuel from Biomass” (TMFB) of the German Research Foundation (DFG), ecotoxicological bioassays were applied for the first time as part of the development process of novel biofuel molecules. These first experiments should give insight into required modifications of the bioassay protocols, and deliver a first assessment of the ecotoxicological hazard potential of these potential biofuels. Moreover, as part of an environmentally conscious and sustainable design process within the TMFB, they allow the identification of potential harmful fuel candidates at an early stage of the development and can be useful for an early selection of low hazard biofuel candidates for further development.[27] *In vitro* cytotoxicity tests are commonly applied as rapid and reproducible biotests[42–46] that can be used in a preliminary screening for the identification of toxic samples, or for defining required modifications of the test design. They can give a first insight into the acute toxic potential of a sample and are often applied prior to mechanism-specific bioassays. In this regard, the Neutral Red retention (NR) assay is ideally suited for this study and the investigation of nearly unknown substances, such as biofuel candidates, due to its relatively simple and easily modifiable test design. It is commonly applied for the detection of cytotoxic effects before investigating a sample in mechanism-specific bioassays.[31,47,48] These assays, such as the EROD assay, give insight into the toxic mechanisms of the investigated biofuel samples, such as the induction of the aryl-hydrocarbon receptor (AhR). These cellular changes that can be detected by the EROD assay are often first warning signals for an environmental disturbance.[49] Both bioassays were applied using the liver-derived cell line RTL-W1 (*Oncorhynchus mykiss*), which is considered as a sensitive tool for the investigation of the toxic potency of environmental contaminants or mechanism specific endpoints, such as EROD activity.[30,50]

Many studies identified levulinic acid, hydroxymethylfurfural or furfural as very promising platform chemicals for future fuel production.[51–54] In the present study, three promising substances with good combustion properties, ethyl levulinate (EL), 2-methyltetrahydrofuran (2-MTHF) and 2-methylfuran (2-MF)[55–57] were investigated for their hazard potential. To allow a comparison to the fossil fuels they are supposed to replace, their ecotoxicity was compared to a fossil gasoline fuel (G), a fossil diesel fuel (D) as well as an established biodiesel (BD; rapeseed oil methyl ester, RME).

The aim of this study was to investigate the Neutral red retention and EROD activity of potential biofuels as well as identify essential modifications of *in vitro* tests systems for the investigation of the toxicological potencies of biofuels by means of the NR assay and the EROD assay. Furthermore, we want to compare toxicological effects of these potential biofuels to those of established reference fuels. For the investigation of these reference fuels, a WAF approach and a passive dosing approach with silicone O-rings were compared.

## Material & Methods

### 2.1 Fuel samples

The three biofuel candidates EL, 2-MTHF and 2-MF were purchased from Sigma-Aldrich with a purity of at least 99%. More information are shown in Table 1.

**Table 1:** Overview of the potential biofuels.

Biofuel	CAS#	LogKow*	Solubility [g/L]	Vapour pressure [mm HG]
Ethyl levulinate	539-88-8	0.288	152[58]	0.208**
2-Methyltetrahydrofuran	96-47-9	1.354	140[59]	97.344**
2-Methylfuran	534-22-5	1.910	3**	156.25**

*estimated using EPI suite Kowwin v1.68

**SRC Physprop database, August 2016

Diesel fuel (ultra low sulphur diesel) and gasoline fuel (unleaded) were purchased from Shell Deutschland Oil GmbH (Hamburg, Germany) and RME biodiesel was kindly provided by ADM Hamburg AG (Hamburg, Germany).

### 2.2 Cell culture of the permanent cell line RTL-W1

Cytotoxicity and AhR agonist activity were investigated using the permanent cell line RTL-W1 derived from rainbow trout (*Oncorhynchus mykiss*).[50] Cells were maintained in 75 cm^2^ cell culture flasks in L-15 (Leibovitz; Sigma-Aldrich) medium supplemented with 9% fetal bovine serum (FBS; Biowest, Nuaillé, France) and 1% penicillin/streptomycin solution (Pen/Strep, with 10,000 units penicillin and 10 mg streptomycin per ml in 0.9% NaCl; Sigma-Aldrich). RTL-W1 cells were kept in darkness at 20°C. Cells were passaged once a week in a ratio of 1:2 using 1x trypsin/EDTA (Sigma-Aldrich).

### 2.3 Preparation of the reference fuels: WAFs & passive dosing

Cytotoxicity of the reference fuels was investigated using water accommodated fractions (WAFs) and a passive dosing approach. For investigations of AhR agonism, only WAFs were used. WAFs for each reference fuel were prepared by low energy mixing (overhead shaker; Reax 20, Heidolph Instruments GmbH & Co. KG, Schwabach, Germany) in 1 L amber glass bottles (Duran Group GmbH, Wertheim/Main, Germany) with a headspace volume of approximately 135 ml. 1–100 g/L gasoline (G-WAF), 0.01–100 g/L diesel (D-WAF) and 100 g/L biodiesel (BD-WAF) were prepared. Different concentrations were prepared directly and not by dilution of the highest concentration, since the petroleum hydrocarbon concentrations do not change linearly.[60,61] After mixing for 24 h the suspensions were transferred into 1 L separation funnels and allowed to settle overnight before draining the WAFs from the separation funnels. Additionally, a process control (ProCo) consisting of 1 L ultrapure water was prepared identically. An overview of the generated WAFs is given in Table 2. The concentrations 100 g/L diesel, 100 g/L biodiesel, as well as 50 g/L gasoline were tested in three replicates in both bioassays. Lower concentrations were tested in fewer replicates, since no effects were detected in higher concentrations.

**Table 2:** Number of independent replicates and concentrations for each generated WAF.

Fuel amount per 1000 ml water [g]	Gasoline	Diesel	Biodiesel
100	1	3	3
50	3	-	-
25	2	-	-
12.5	2	-	-
6	1	-	-
3	1	-	-
1	1	-	-
0.1	-	2	-
0.01	-	1	

For passive dosing, loaded silicone O-rings were placed in the wells of a 96 well plate, where they served as donor for maintaining exposure throughout the test.[62,63] O-rings with an outer diameter of 6.46 mm (Altec Products Limited, Cornwall, UK) were pre-cleaned by soaking once overnight with ethyl acetate (>99%; Sigma-Aldrich) and three times, each overnight, with methanol (>99%; Sigma Aldrich). To completely remove methanol from the rings, they were washed three times, each overnight, with water.[62] They were then loaded by direct immersion within a loading solution (Table 3) consisting of a sample (G, D or BD) and virgin olive oil (manufactured mechanically, without additives) as the diluent at a ratio of 6 O-rings per 4 ml loading solution at 200 rpm and 20°C for 72 h. As a process control, O-rings were loaded with olive oil. After loading, O-ring surfaces were thoroughly wiped dry using a lint-free tissue paper.

**Table 3:** Overview of the loading solutions applied for the loading of the O-rings. D = diesel, G = gasoline, BD = biodiesel

Fuel concentration in the loading solution	Fuel tested	Volume fuel [ml]	Volume olive oil [ml]
100 %	D,BD	4	0
50 %	G, D, BD	2	2
25 %	G, D	1	3
12.5%	G, D	0.5	3.5
Process Control	-	0	4

The loaded O-rings were used to pre-equilibrate the L-15 medium (3 rings/2 ml) under test conditions for 24 h. The loaded rings were also used for exposure control throughout the test. The loading with diesel (D100, D50, D25, D12.5), gasoline (G50, G25, G12.5) and 100% biodiesel (BD100) had made the O-rings swell, and these swelled O-rings could be fitted in the wells without touching the bottom of the well. O-rings loaded with 50% biodiesel (BD50) were also tested with short wires (BD50-wire) placed at the bottom of each well to prevent a direct cell contact of the BD-loaded O-rings and the cells.

### 2.4 Cytotoxicity: Neutral Red Retention (NR) assay

Cytotoxicity was investigated by means of the NR assay according to Borenfreund[64,65] and modifications by Keiter et al.[42] and Klee et al.[66] Further modifications were required depending on the investigated sample and are stated below.

The plate layout was identical for each sample. The outer wells of the plates were used as cell-free and medium-free blanks to measure the background fluorescence intensity. Six negative control wells containing only L-15 medium were placed on both sides of the samples on each plate, respectively, resulting in a total of 12 negative control wells. As a positive control, 40 mg/L 3,5-dichlorophenol (DCP) was tested in six wells.

#### 2.4.1 Exposure with biofuels

For investigation of the potential biofuels EL, 2-MTHF, and 2-MF, seven test concentrations were prepared in glass test tubes by diluting the substances in supplemented L-15 Leibovitz medium and transferred in six replicates on a 96-well plate. Sample dilutions in glass tubes were prepared 2x-concentrated with consideration of later dilution with cell suspension. Final test concentrations are shown in Table 4. Due to their aggressiveness towards plastics, investigation of 2-MF and 2-MTHF took place in 96-well glass plates (Hellma Analytics, Müllheim/Baden, Germany), while EL was tested in a 96-well plastic (polystyrene) plate (TPP Techno Plastic Products, Trasadingen, Switzerland). No differences in cell growth or cytotoxicity of EL were observed in preliminary experiments comparing glass and plastic plates (S1 Fig). Moreover, Hamilton syringes were used instead of microliter pipettes with plastic tips. EL was tested in three independent replicates; 2-MTHF and 2-MF were tested in four independent replicates.

**Table 4:** Final nominal test concentrations in [g/L] of the potential biofuels in the NR assay using RTL-W1 cells.

Substance	Conc. 1	Conc. 2	Conc. 3	Conc. 4	Conc. 5	Conc. 6	Conc. 7
EL	25.400	12.700	10.160	7.620	5.080	2.540	1.016
2-MTHF	43.000	21.500	10.750	5.375	2.687	1.343	0.672
2-MF	2.730	1.365	0.683	0.341	0.171	0.085	0.043

#### 2.4.2 Exposure with reference fuels

Reference samples were prepared as described above. WAFs were supplemented with 13.8 g/L L-15 Leibovitz medium (powder, Sigma-Aldrich), 9% FBS and 1% penicillin/streptomycin solution and were added to 96-well plastic plates in six replicates with 175 μl per well. In consideration of a further dilution due to the addition of cell suspension, the initial WAFs were diluted by 21.25% and, thus, resulting in a dilution of 78.75% of the initial WAF. All WAFs were tested in twelve technical replicates. The process control was tested in six technical replicates. In total, three independent replicates have been conducted.

For passive dosing, 12 loaded O-rings of each sample concentration and 150 μl of pre-equilibrated L-15 medium were transferred into 12 wells of a 96-well plate (~2 h after seeding of the cells) For the process control, 6 O-rings and 6 wells were used. Diesel and biodiesel were tested in a 96-well plastic plate, whereas gasoline was tested in a 96-well glass plate, due to gasoline-loaded O-ring damaging the well-surface. Negative and positive controls were applied as described for the potential biofuels. Each passive dosing concentration with the exception of BD50-wire was tested in three independent replicates. Only one replicate (six technical replicates) of BD50-wire has been tested to confirm the influence of the direct cell-ring contact on cell viability.

#### 2.4.3 Cell seeding

For cell seeding, confluent RTL-W1 cells in cell culture flasks were trypsinized by using 1x Trypsin (Sigma-Aldrich). To attain a final volume of 200 μl per well and a final cell concentration of 2 x 10^5^ cells per ml in each well, 100 μl of a cell suspension adjusted to 4 x 10^5^ cells per ml were transferred to each well containing a potential biofuel, 50 μl of a cell suspension adjusted to 8 x 10^5^ cells per ml were transferred to each well later containing a loaded O-ring, and 25 μl of a cell suspension adjusted to 16 x 10^5^ cells per ml were transferred to each well containing a WAF of a reference fuel. To reduce diffusion of substances to adjacent wells, plates were sealed with a membrane (clear adhesive polyester sealing tape; Thermon Electron LED GmbH, Langenselbold, Germany). Cells were exposed for 48 h at 20°C.

#### 2.4.4 Evaluation and data treatment

After exposure, medium and O-rings were discarded and cells were incubated for 3 h with a 0.005% Neutral red (3-Amino-7-dimethylamino-2methylphenanzine, Sigma-Aldrich) solution. After washing and dye extraction the absorbance of incorporated Neutral red was photometrically measured for determination of cell viability at a wavelength of 540 nm and a reference wavelength of 690 nm using a multimode microplate reader (TECAN infiniteM200; Tecan Austria GmbH, Grödig, Austria).

For data treatment, the mean of the blanks was subtracted from each value and the median of the first negative control was considered as 100% cell viability. A test was defined valid if the median of the viability of both negative controls did not vary by more than 20%. For EL, 2-MF, and 2-MTHF, concentration-response curves were fitted with a nonlinear ‘log(agonist) vs. response–Variable slope’ regression (Eq 1) using GraphPad Prism 6.02 (GraphPad Inc., San Diego, USA).

$$x=C-(log10\frac{\left(B-A\right)}{\left(y-A\right)}-1)/D$$ (1)

where x is the concentration, y is the percentage of cell viability, A is the Bottom plateau value, B is the Top plateau value, C is the LogEC_50_, and D is the unitless Slope factor of the curve. Concentrations resulting in cell viability of 50% and 80% were calculated (NR_50/80_-values) for each replicate. Furthermore, hydrocarbon (HC) concentrations of the biofuel NR_50_-values were assumed by calculating the percentage of hydrogen and carbon from the NR_50_-value. Subsequently, the mean of the three or four NR_50_-values for each substance was calculated. For the reference fuels, cell viability of the controls and each sample was normalized against the first negative control.

### 2.5 AhR agonist activity: EROD assay

The investigation of AhR agonist activity, so called dioxin-like activity, was performed according to Behrens et al.[67] using the EROD assay with modifications published by Seiler et al.[44] Prior to the beginning of exposure RTL-W1 cells were seeded in 96-well plates and allowed to grow confluent at 20°C for 72 h to prevent increased background EROD induction due to stress. 2-MF and 2-MTHF were tested on 96-well glass plates and EL was tested on 96-well plastic plates. Preliminary experiments revealed no differences of the cells in sensitivity for a TCDD standard (S2 Fig). For the potential biofuels, eight test concentrations were prepared in glass test tubes according to Table 5. As highest test concentration, the NR_80_-value as determined by means of the NR assay was applied.[31,47,48] WAFs were supplemented with 13.8 g/L L-15 Leibovitz medium (powder, Sigma-Aldrich), 9% FBS and 1% penicillin/streptomycin solution, thus resulting in a dilution of 90% of the initial WAF. Each sample was tested in three independent replicates and each sample concentration was tested in six technical replicates per plate.

**Table 5:** Nominal test concentrations in [g/L] of the potential biofuels in the EROD assay using RTL-W1 cells.

Substance	Conc. 1	Conc. 2	Conc. 3	Conc. 4	Conc. 5	Conc. 6	Conc. 7	Conc. 8
EL	7.112	3.556	1.829	0.914	0.406	0.203	0.102	0.051
2-MTHF	10.750	5.375	2.688	1.344	0.672	0.336	0.168	0.084
2-MF	0.546	0.455	0.364	0.273	0.182	0.091	0.046	0.023

As a positive control, 2,3,7,8-tetrachlorodibenzo-*p*-dioxin (TCDD; Sigma-Aldrich) was serially diluted (3.125–100 pM) in two replicates in L-15 medium. After discarding the growth medium, controls and samples in medium were added to the 96-well plates in 200 μl per well. Cell exposure was terminated after 72 h (for optimum concentration-response curves according to Gustavson et al.[68]) by discarding the exposure medium and freezing of the cells at -80°C for at least 1 h. Measurement of EROD induction was performed as described by Heger et al.[41] Frozen 96-well plates were thawed and 100 μl 7-ethoxyresorufin and 50 μl NADPH (1.2 μM and 90 μM, respectively, in phosphate buffer) were added to each well. Subsequently, after 10 min, EROD reaction was terminated by addition of 0.54 mM fluorescamine in acetonitrile. Resorufin measurement was conducted at an excitation wavelength of 544 nm and emission of 590 nm using a multimode microplate plate reader (TECAN infiniteM200; Tecan Austria GmbH, Grödig, Austria). Measurement of fluorescamine-bound protein took place at an excitation wavelength of 355 nm and an emission at 460 nm. EROD induction was determined as pmol resorufin x mg protein^-1^ x min reaction time^-1^ and normalized to the negative control.

### 2.6 Statistical Analyses

Shapiro Wilks test for testing of normality was performed for all samples using SigmaPlot 12.0 (Systat Software Inc., Chicago, USA). One Way ANOVA with Dunnett’s post-hoc test (with Browns-Forsythe Test for testing of homoscedasticity) was applied for detection of significant cytotoxic effects of the biofuels and the reference fuels as well as significant differences between NR_50_-values of the three potential biofuels in the NR assay. Calculations were performed using GraphPad Prism 6.02. Kruskal-Wallis One Way ANOVA on ranks was performed for detection of significant increased EROD induction of the biofuels and the reference fuels using SigmaPlot 12.0.

### 2.7 Chemical analyses

The dissolved organic carbon (DOC) concentrations of WAFs used for NR assay and EROD assay were measured. Therefore, ~10 mL of each sample were filtered (PALL, Acrodisc IC, 0.45 μm PES membrane) and the pH was adjusted to <2. Measurement was performed at the Institute for Hygiene and Environmental Medicine of the RWTH Aachen University. For pre-equilibration experiments with the passive dosing tests, gas chromatography with flame ionisation detector (GC-FID) analysis was performed due to the smaller sample volume available compared to the WAFs. The larger sample volume of the WAFs allowed a measurement of the DOC content. O-rings were extracted directly using 10 ml n-hexane (C. Roth, Karlsruhe, Germany). Extracts were evaporated using a rotary evaporator and analysed by means of GC-FID (7820A GC System, Agilent Technologies). For calibration, an alkane standard (C8, C10, C16, C28) was measured. A 30 m HP-5 GC column with a 320 μm ID and a 0.25 μm film was used. The oven program was 45°C for 3 min and 8°C/min to 275°C which was held for 7 min. Carrier gas (helium) flow rate was held at 1.5 ml/min and the detector temperature was fixed at 340°C. Air and H_2_ were used as combustion gases at flows of 400 and 30 ml/min, respectively. Chromatograms were analysed by means of baseline integration from C10 to C28 using EZChrom Elite Compact (ver.3.3.2, Agilent Technologies).

Chemical analyses of the biofuels were performed in water (GPR Rectapur®, VWR International GmbH, Darmstadt, Germany). For the investigation of losses six wells of a 96-well plate were filled with 200 μl of a sample concentration. After 48 h, the volume of the six wells was pooled for analyses using HPLC with diode-array detector (Agilent technologies 1200 series equipped with G1315C DAD SL). For biofuel analyses, 40 μL samples were injected directly at 25°C (EL) and 40°C (2-MTHF & 2-MF). Samples were separated on a 125 x 4 mm LiChrospher 100 RP 8 EC– 5μ column (CS Chromatographie Service, Langerwehe, Germany), operated at a flow rate of 1 mL min^-1^. As mobile phase, methanol and water were used. HPLC gradients were used as described by Bluhm et al.[69]

## Results

### 3.1 Cytotoxicity

#### 3.1.1 Biofuels

**Fig 1:**
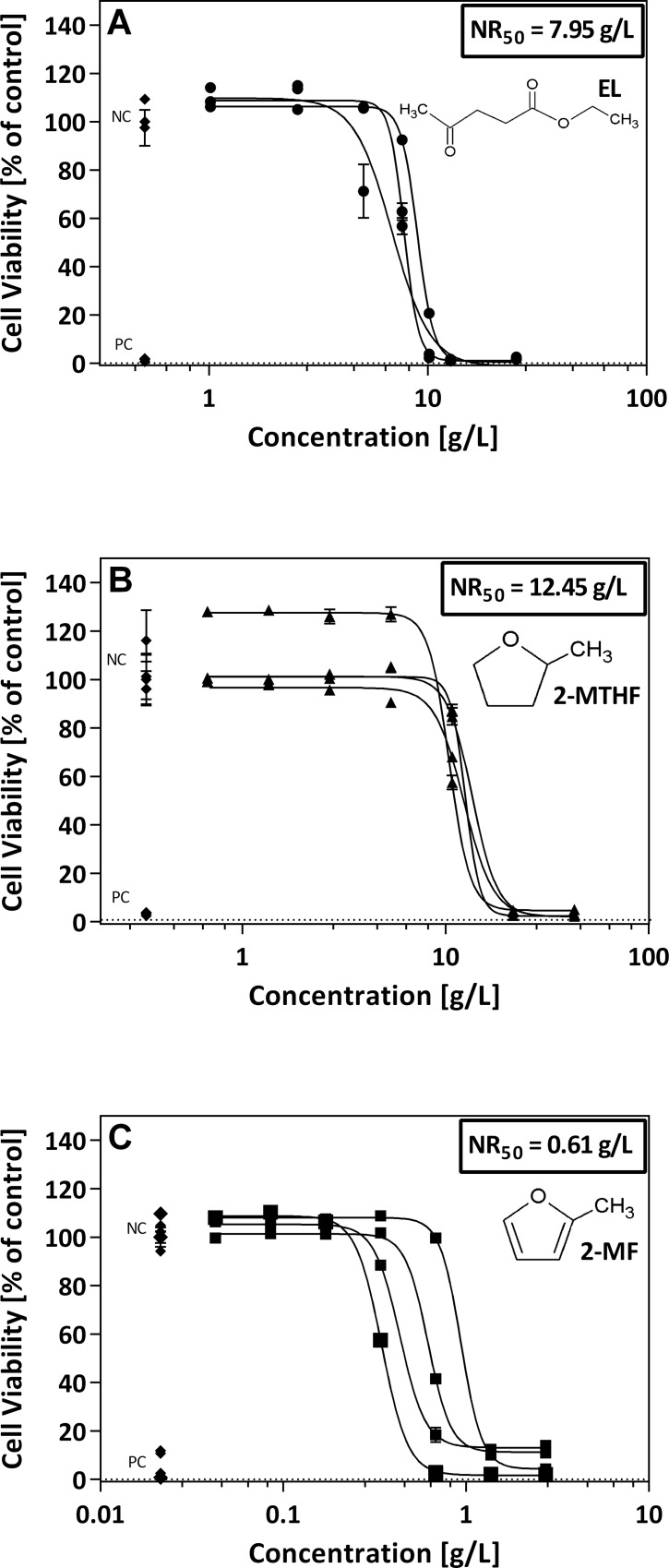
**Cytotoxic effects on RTL-W1 cells caused by EL (A), 2-MTHF (B), and 2-MF (C) determined by the Neutral red retention assay.** Data are given as means (dots) and standard deviation (SD; error bars). The x-axes give the nominal concentrations. Each curve represents one independent replicate. The numbers show the NR_50_-value for the respective sample calculated as the mean of the three or four NR_50_-values determined for each replicate. Lower NR_50_-values indicate a higher cytotoxic potential. n_EL_ = 3, n_2-MF,2-MTHF_ = 4

The NR assay revealed significant differences in the cytotoxic potential between the substances. The NR_50_-values (concentrations with 50% cell viability, lower NR_50_-values indicate higher cytotoxic potential) differed significantly (p<0.05) by a factor of ~13 between 2-MF and EL and a factor of ~1.6 between EL and 2-MTHF. 2-MF showed the highest cytotoxic potential with an NR_50_-value of 0.61 g/L, while testing of EL and 2-MTHF revealed NR_50_-values of 7.95 g/L and 12.45 g/L, respectively (Fig 1).

In Table 6 nominal concentrations, measured initial concentrations and measured concentrations after 48 h of two concentrations are shown that are enclosing the NR_50_-value.

**Table 6:** Overview on losses for each biofuel candidate. Shown concentrations are initial concentrations (t = 0h) and concentrations after t = 48 h (incubation period of the NR assay). Concentrations were chosen according to the EC_50_-value of the biofuels. Substances were analysed under test conditions in 96-well plates diluted in water without cells. n = 1

Substance	Concentration t = 0h [g/L]	Concentration t = 48h [g/L]
2-MF	(0.68)^a^ 0.625	0.208
	(0.34)^a^ 0.294	0.107
EL	(12.7)^a^ 12.784	12.751
	(10.16)^a^ 10.168	10.125
2-MTHF	(10.75)^a^ 11.115	3.781
	(5.38)^a^ 5.502	2.180

^a^nominal concentration

Effective chemical activities for the NR assay (EA-50_NR_) were calculated for each compound according to Schmidt and Mayer (2015)[70] by dividing by the respective EC_50_-value (Fig 1) with the water solubility (Table 1). For each substance, the EA-50_NR_ is well above 0.01, with EL showing a lower EA-50_NR_ (0.052) than 2-MTHF (0.089) and 2-MF (0.203).

#### 3.1.2 Reference fuels

WAFs and passive dosing investigations of the reference fuels revealed different results for WAFs in comparison to the passive dosing approach. WAFs did not induce a cytotoxic effect in the highest concentrations (G-WAF: 50 g/L; D-WAF and BD-WAF: 100 g/L; S3 Fig). Cell viability varied within 5% compared to the negative control and was well within the 20% limit of test validity.

DOC measurement (for the undiluted WAFs!) revealed 1390 mg/L DOC in G 50 g/L, ~3 mg/L in D 100 g/L and ~8 mg/L in BD 100 g/L. For the process control, DOC concentrations were below 1 mg/L (Table 7).

**Table 7:** DOC concentrations of gasoline, diesel and biodiesel WAFs. The concentrations are shown in [mg/L]. n_Diesel, Biodiesel_ = 2, n_Gasoline_ = 1

WAF	Concentration (DOC) [mg/L]	
Gasoline 50 g/L	1390.8	
Gasoline 12.5 g/L	325.5
Diesel 100 g/L	3.3±0.5
Diesel 0.1 g/L	1.5±0.0
Biodiesel 100 g/L	8.2±0.1
Process Control	1.0±0.3

**Fig 2:**
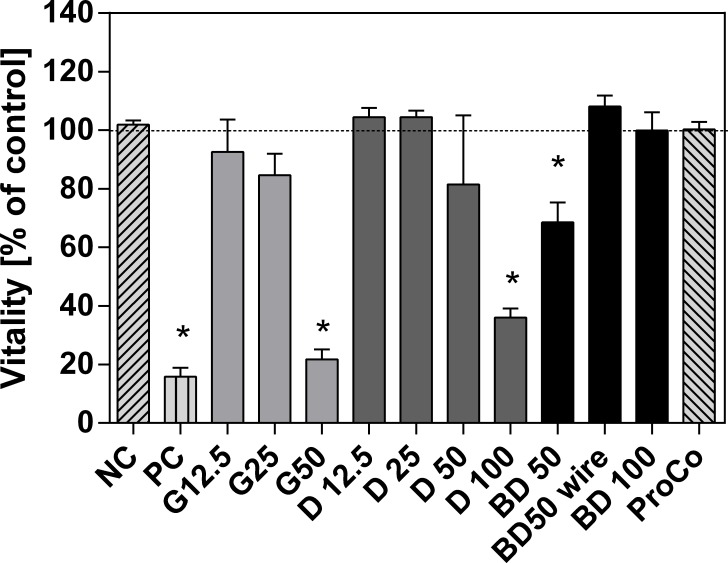
Cytotoxic effects on RTL-W1 cells caused by G, D, and BD in a passive dosing approach. Data are given as means (bars) and SEM (error bars) of the cell viability [%] of the negative controls. Numbers represent the concentration of a fuel the O-rings were loaded with (e.g., 25: 25% fuel, and 75% olive oil). The dotted line represents 100% cell viability. Asterisks denote significant differences in comparison to the negative control (p<0.05, One Way ANOVA with Dunnett’s post-hoc test). n_BD50wire_ = 1, n_G,D,BD_ = 3, n_ProCo,PC_ = 10, n_NC_ = 10

The passive dosing approach revealed significant cytotoxic effects for BD50, G50, as well as D100 (Fig 2). Highest cytotoxicity was found for the highest gasoline concentration (G50: 21.69% ± 2.20% cell viability) followed by the highest diesel concentration (D100: 45.91% ± 6.92% cell viability). BD50 tested without wire (see Materials and Methods) revealed significant cytotoxic effects (BD50: 70.43% ± 7.69% cell viability), even though the highest biodiesel concentration BD100 showed no cytotoxic effects. However, when tested with wire to prevent cell-ring contact, BD50 revealed no cytotoxic effects.

### 3.2 AhR agonist activity: EROD assay

Neither the potential biofuels nor the WAFs of the reference fuels revealed any significant dioxin-like activity. EROD induction did not exceed 2.02 ± 0.80 fold induction of the negative control for any of the biofuel samples and 1.83 ± 0.79 for the WAFs of the reference fuels (S4 Fig and S5 Fig). Passive dosing was not investigated in the EROD assay.

## Discussion

### 4.1 Modifications of the Bioassays

First experiments with the NR assay revealed several challenges regarding the exposure that have to be considered for the testing of biofuels as well as fossil fuels. Material incompatibility, test substance volatility, and, in particular for the reference fuels, hydrophobicity were identified as potential obstacles and could possibly affect the results of ecotoxicological biotestings, e.g. by giving false positive results or contaminating controls.

While no additional measures, such as the application of solubilizers, were needed for biotesting of the three potential biofuels, the reference fuels required pretreatment steps to enable testing in bioassays. Fossil gasoline and diesel fuels consist of a mixture of known and unknown components with differing water solubility and Henry’s Law behaviours,[60] which complicates the generation of a stable solution or concentration in an aqueous test medium. For overcoming these challenges, the use of WAFs or the application of a passive dosing approach is possible.[60,71] Test results demonstrated that passive dosing is an advantageous tool for investigation of complex fuel samples due to a more reliable dosing of the petroleum hydrocarbons. However, physical impact of the dosing system on the test system should be prevented.

For biotesting, material compatibility was identified as an important preliminary requirement of biomass-derived and fossil fuels. Thus, plastic components were replaced with alternative glass or steel components, e.g., Hamilton syringes and 96-well glass plates. Neither cell growth nor EROD induction were found to be impeded by the glass plates (S1 Fig and S2 Fig). Besides 2-MTHF and 2-MF, gasoline revealed an increased aggressiveness towards plastics. Precisely, gasoline-loaded O-rings were found to corrode the inner well surface when in direct contact with plastic plates. Therefore, gasoline was also tested in 96-well glass plates. However, this observation was not made for any of the G-WAFs. Since well surface corrosion was only observed at direct contact between O-ring and plastic, it was assumed that the corroding components are hydrophobic compounds that remained in the O-ring in high concentration and did not solve into the aqueous medium. Moreover, the direct contact between O-ring and plastic enables a direct diffusion of gasoline hydrocarbons in the plastic without any indirect route through the aqueous phase. This could probably accelerate and reinforce the deformation of the plastic.

A further potential impact on the results of potential biofuels and fossil fuels is their volatility. 2-MF and 2-MTHF are volatile organic compounds (VOC).[72] Observations in preliminary cytotoxicity tests with 2-MF and 2-MTHF indicate that volatilization might lead to cross contamination between compounds in adjacent wells or a contamination of the controls (observed change of the medium colour in negative control wells next to the highest samples concentration). Therefore, test vessels had to be membrane sealed during testing. However, the blistering of the membrane and the formation of air pockets could not be entirely prevented, and thus very small amounts of test compounds could evaporate in, e.g., some control wells. The concentration of this cross contamination, however, was below the limit of detection (LOD) for HPLC analyses. It was assumed with respect to the appearance of small visible peaks in the negative control adjacent to the highest 2-MF concentration after 48 h at the same retention time identified for 2-MF (S1 File, S2 File). Thus, this cross contamination was not considered to significantly affect the test system. However, sealing by a membrane did not prevent losses of some potential biofuels from the 96-well plate, in particular of 2-MF or 2-MTHF. Only ~10–35% and ~30–40% of the initial test concentration could be found in the aqueous phase after 48 h exposure in a 96-well plate in a blank experiment for 2-MF and 2-MTHF, respectively (Table 6). The high volatility of 2-MF and 2-MTHF (Table 1) corroborates that most losses occur by evaporation. Therefore, a better sealing of the test vessels has to be established. However, losses of EL were below 1% and considered to be negligible.

Since similar issues were expected for investigation of the reference fuels, test plates containing the reference fuels were sealed as well. In particular, the complex fossil gasoline fuel contains a large number of volatile low molecular (<C_10_) hydrocarbons, and rapidly declining petroleum hydrocarbon concentrations in the WAF experiments due to volatilization[73] are assumed to be the main reason for the lack of any cytotoxic effects. However, passive dosing can maintain a constant concentration of these components, because the reservoirs (silicone O-rings) can continually compensate the losses of these volatile molecules as long as they are not depleted.

### 4.2 Cytotoxicity of biofuels

Investigation of the three potential biofuels revealed significant differences in the cytotoxic potencies. A factor of ~20 between the NR_50_-values of 2-MF, the most cytotoxic substance, and 2-MTHF, the least cytotoxic compound, demonstrate the variance in concentration ranges that have to be considered for the testing of different biofuels (Fig 1). However, by calculating and comparing the EA-50_NR_ values, this factor is reduced to ~4. All EA-50_NR_ values were well above 0.01 and, thus, indicating a baseline toxicity and no excess toxicity. According to Reichenberg et al.[74], baseline toxicity requires a chemical activity of at least 0.01–0.1, whereas a EA-50 value well below 0.01 would suggest a specific mode of toxic action. However, since the EC_50_-values for the potential biofuels were calculated from nominal concentrations, they are probably overestimating the actual EC_50_-values (and the EA-50_NR_ values) due to the high volatility of, e.g., 2-MF and 2-MTHF.

Unfortunately, little ecotoxicological data is available on these three substances, even though each substance has already been applied as a fuel additive[55,56,58,75] and EL is also used as a flavour at very low concentrations.[76] However, since many furans, such as furan, tetrahydrofuran, dibenzofuran or menthofuran, are known to cause adverse effects on lung, liver, and kidney tissue,[77–82] toxic effects, in particular induced by alkylated furan such as 2-MF, were expected.[81] In case of 2-MF, enzymatic bioactivation by a cytochrome P450 monooxygenase to the main intermediate acetylacrolein (AA) appears to be the major mechanism of its toxicity.[83–86] The intermediate AA was found to bind rapidly to tissue macromolecules, such as microsomal proteins[83,84] that are involved in many vital functions of the metabolism, e.g., the metabolization of endogenous and exogenous substances.[87]

**Fig 3:**
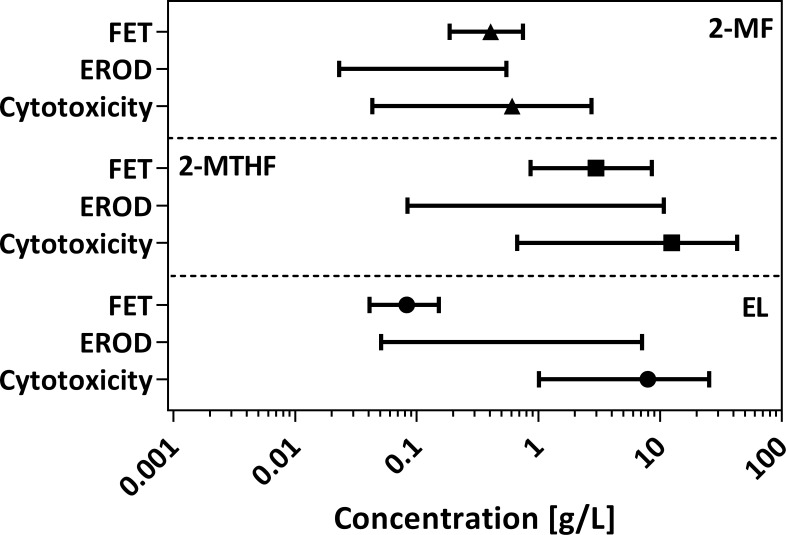
Overview on NR_50_/LC_50_-values (symbols) and concentration ranges (bars) for the three potential biofuels 2-MF, 2-MTHF and EL for NR assay (cytotoxicity) and EROD assay (dioxin-like activity) and the fish embryo toxicity test (teratogenicity/embryotoxicity; (69)). The lack of symbols indicates that no EC_50_ value could be obtained.

Information on the processes involved in the toxicity of 2-MTHF and EL are less prevalent. There are few studies on the toxicity of 2-MTHF and it is considered generally nontoxic to humans.[88,89] With an NR_50_-value of 7.95 g/L, EL also appears to have a similar low toxic potential as 2-MTHF (NR_50_-value 12.45 g/L). Since toxicity of derivates of levulinic acid, such as EL, often depends on the length of the alkylated side chain and their hydrophobicity, EL is considered slightly more toxic than the parent compound levulinic acid.[90] However, as could be shown by Bluhm et al.,[69] the toxic potential of EL (LC_50_: 0.083 g/L) in the fish embryo toxicity test is significantly increased compared to 2-MF (LC_50_: 0.405 g/L) and 2-MTHF (LC_50_: 2.98 g/L) (Fig 3). For EL and 2-MTHF, acute embryotoxicity was found to be nearly two orders of magnitude and approximately 4 times higher than cytotoxicity, respectively. For 2-MF, no differences could be detected. *Danio rerio* embryos appear to be very sensitive to an exposure with EL. However, the specific mode-of-action is not known and, thus, the difference in the relative toxicity of the three biofuel candidates cannot be completely explained. Moreover, many fish cell lines are known to be several orders of magnitude less sensitive than fish.[91] The increased toxicity of EL to *D*. *rerio* is most likely caused by species-specific differences between *D*. *rerio* embryos and RTL-W1 cells or differences between the level of complexity (cell vs. embryo). Applying a different and more sensitive test system, such as the RTgill-based toxicity assays proposed by Tanneberger et al.[91], might result in an effect concentration more similar to the LC-value determined for *D*. *rerio* embryos. Thus it could be examined if other cell line than RTL-W1 would be favourable for a screening regarding the cytotoxic potential of biofuel candidates in the future.

These findings highlight once more that extrapolating toxic potencies even for the baseline toxicity of newly investigated substances on, e.g., different organisms or different levels of organisation (cell culture vs. whole organism) is limited. Therefore, further aquatic bioassays with different test organisms and also different levels of complexity have to be considered.

### 4.3 Cytotoxicity of fossil fuels: WAFs & passive dosing

Gasoline and diesel fuel are known to contain a high amount of low molecular weight substances, such as monoaromatic hydrocarbons (BTEX: benzene, toluene, ethylbenzene, xylene) and polycyclic aromatic hydrocarbons (PAHs),[5,6] which are known to be highly (cyto)toxic.[92,93] Sulphur, nitrogen and fluor-containing compounds are also present in fossil fuels, but due to restrictive regulatory practises, these components are usually removed, e.g. by the use of ionic liquids.[94] Due to the sample composition, significant differences between the fossil fuels and the RME biodiesel were expected, with gasoline showing stronger toxic effects than diesel because of a higher concentration of these toxic aromatic hydrocarbons.[6,32] Cytotoxicity of fossil fuels is reported to be caused by non-specific, narcotic effects.[95–97] According to Van Wezel et al.[98] this non-specific toxicity is the result of an increased fluidity of the cell membrane after a certain critical body residue (CBR) is attained.

The lack of biodiesel-WAF cytotoxicity was in good concordance with biodiesel-WSF simulation experiments conducted by Leme and co-workers.[99] A similar lack of cytotoxicity was expected for the passive dosing testing of RME biodiesel. However, no comparable studies were available. Therefore, the significant cytotoxic effect observed for the lower biodiesel concentration tested in the NR assay was unexpected. It was assumed to be induced by a direct contact between the loaded O-ring and the cells. Due to a lack of O-ring swelling as observed for gasoline (G50, G25, G12.5), diesel (D100, D50, D25, D12.5) and BD100, BD50-loaded O-rings were lying directly on the cells. Whereas a direct contact between olive-oil loaded O-rings (= the process control, no O-ring swelling was observed) and cells had no negative effects, BD50-loaded O-rings might transfer fatty acid methyl esters from the O-ring into the cell membrane. Thus, membrane composition and fluidity would be changed leading to functional restriction of the membrane.[98] For verification of this assumption, further experiments were conducted with BD50-loaded O-rings and short wires (to prevent direct contact of the rings with the cells). By this, we ensured that cytotoxic effects could have been solely induced by compounds partitioned into the water phase. First results indicated that the water soluble compounds are not responsible for the cytotoxic effect of BD50. Thus, the direct ring-cell-contact might have induced the observed effect. However, further replicates are required to support this hypothesis.

The differences between the cytotoxicity of diesel and gasoline by the WAFs and the passive dosing approach can be explained by their different hydrocarbon concentrations and composition in the test media. First available results revealed the hydrocarbon concentration of diesel (~12 mg/L) for O-rings loaded with 100% diesel in pre-equilibrated medium at the beginning of the NR assay in the passive dosing approach, which is ~4 times higher than hydrocarbon concentrations in D-WAFs (Table 7). These differences in hydrocarbon concentrations may be due to the different compositions of fuel components in the passive dosing and WAF approach. WAFs contain mainly water-soluble, volatile constituents,[60] whereas the pre-equilibrated passive dosing medium is assumed to contain more hydrophobic, long-chained petroleum hydrocarbons in higher concentrations. This would also explain why cytotoxicity was not induced by G-WAF: The petroleum hydrocarbon concentration in the 50 g/L G-WAF (1.39 g/L) decreased very rapidly after the WAF generation to a very low level that induced no cytotoxicity. Based on our results, the passive dosing appears to be advantageous regarding investigations of fossil fuels. Not only the volatile compounds can be partially replaced but also a supply with hydrophobic compounds over the test duration is improved.

Since none of the WAF induced cytotoxicity, only results for the passive dosing approach can be used for a comparison to the cytotoxic potencies of the three potential biofuels. D100 was found to induce 50% reduced cell viability at a measured pre-equilibrated concentration of ~12 mg HC/L at the on-set of the NR assay (data not shown), which is at least a factor ~40 lower than the most cytotoxic potential biofuel 2-MF (NR_50_-value: 490 mg HC_calculated_ /L). However, keeping in mind the high losses of 70–90% after 48 h, this NR_50_-value is certainly underestimating the actual cytotoxic potential of 2-MF. Therefore, even though the potential biofuels appear to be less cytotoxic than the fossil reference fuels, for a detailed comparison of different fuels, sufficient data on the behaviour of the test substances in the test medium has to be available. In this regard, more comprehensive chemical analyses are very important, e.g. to confirm constant exposure of the cells in the passive dosing approach at the saturation level of the reference fuels.[100]

### 4.4 AhR agonist activity: dioxin-like activity

Our results showed that no dioxin-like activity was caused by the investigated biofuels. The reason may be due to the molecular structure of these biofuel candidates. Traditional ligands for AhR-induction are described as hydrophobic, planar and/or halogenated aromatic compounds,[101,102] which was obviously not met by the biofuels in this study.

Similar to the EROD results of biofuel candidates, WAFs did not cause any significant EROD induction. The lack of any EROD activity induced by the fossil fuels is probably due to the evaporation of volatile fuel components (see 4.1). Nogueira et al. (2013) also reported no dioxin-like activity during the investigated period of time for biodiesel.[103] However, several other studies reported EROD activity induced by diesel and gasoline.[103–110] In most cases, low molecular and volatile PAHs, such as BTEX and naphthalene, were reported to induce EROD activity,[105,106] while some studies indicate that EROD activity is not exclusively affected by low molecular and volatile PAHs, but also by less volatile fuel compounds,[108] other relativley soluble organic fuel-oil compounds[111] or anorganic fuel components such as sulphur or lead.[112,113]

Differences in EROD activity are most likely due to different concentrations of solved petroleum hydrocarbons in the WAF or the WSF. They can be influenced by the shaking duration, mixing energy and duration until phase separation. But also different test organisms or testing conditions, e.g., static or semi-static exposure/ *in vitro* or *in vivo*, the origin of the investigated fuels[114] or changes in the legal regulations that restrict concentrations of known pollutants (benzene, sulphur etc.)[115] have to be considered for a comparison of different fossil fuels. Therefore, for a sufficient comparison of published data, three main criteria are recommended to be provided: (1) a solid chemical analyses method, (2) sufficient information on the origin of the fuel and (3) if possible information about additives of the fuel.

## Conclusions

In this study, two *in vitro* bioassays were modified to investigate the toxicological potencies of biomass-derived fuel candidates and fossil reference fuels. The testing concentrations of these three biofuels were within their water solubility. Thus, no additional measures such as the application of solubilizers were required. However, for investigation of the reference fuels, pretreatment steps were required to enable testing in bioassays. Passive dosing was found to be advantageous compared to the WAF approach due to a more constant petroleum hydrocarbon concentration in the test medium. Moreover, further improvements are required, such as the prevention of a physical impact on the test system and a reliable method for chemical analyses. These chemical analyses are essential for a comparison of the relative toxic potency of fossil fuels and biofuels.

For both samples, biofuels and fossil fuels, modifications on the test system regarding the high volatility and the material compatibility are required. Evaporation of volatile biofuels or low-molecular petroleum hydrocarbons directly affects the exposure of the test organisms. Sample-induced deformation of the test vessel material might lead to dissolution of material components, such as plasticisers, or physically affect the test system, e.g. by impeding cell growth. For testing of volatile fuels, a closed system is recommended. However, the applied polyester sealing tapes were not sufficient to prevent the evaporation of substances out of the test system according to the results from HPLC. Therefore, different sealing systems, e.g. closed vials with PTFE seal, might be more suitable for the testing of these volatile fuels. Material compatibility was addressed by replacing plastic materials, such as pipette tips and plastic plates, with Hamilton syringes and glass plates, respectively. These modifications did not affect the investigated test systems.

Taking the results from the passive dosing testing as a basis for a comparison of the cytotoxicity of the samples, the biofuels appear to be less cytotoxic than diesel. For a better assessment, more information on the test concentrations of gasoline and diesel in the passive dosing approach are required. Moreover, the differences in dosing methods (passive dosing vs direct dosing) have to be taken in consideration.

## Supporting Information

S1 Fig Preliminary testing of cell growth on glass and plastic plates. Absorption of Neutral red retained from cells grown on one plastic (black) and one glass (grey) plate. Data is shown as the absorption at 540 nm (see 2.4.4) for both negative control rows and the positive control row on each plate (see 2.4).
(TIF)

S2 Fig EROD induction of cells grown on glass and plastic plates. TCDD standard curves (3.125–100 pM) and EC_25_-values obtained by preliminary experiments on a plastic plate (A) and a glass plate (B). Data is shown as the EROD induction in pmol*mg**min^-1^
(TIF)

S3 Fig Cytotoxic effects on RTL-W1 cells caused by WAFs of the reference fuels. Cell viability in [%] of the negative control for the WAFs of gasoline (50 g/L), diesel (100 g/L), biodiesel (100 g/L), the process control (Millipore water) and the positive control (40 mg/L DCP). n_G,D,BD_ = 3, n_ProCo_ = 4, n_NC,PC_ = 6
(TIF)

S4 Fig EROD induction of the three potentialbiofuels. EROD induction in fold induction of the negative control for EL, 2-MTHF and 2-MF. n = 3
(TIF)

S5 Fig EROD induction of the WAFs of the reference fuels. EROD induction in fold induction of the negative control for Diesel and gasoline WAFs. n = 3
(TIF)

S1 File HPLC chromatogram of 1.365 g/L 2-MF in Millipore water after 48 h. (TIF)

S2 File HPLC chromatogram of a negative control (Millipore water) after 48 h. (TIF)
