# Supplementary figures and images for: Microscale In Vitro Assays for the Investigation of Neutral Red Retention and Ethoxyresorufin-O-Deethylase of Biofuels and Fossil Fuels
Source: PLoS One. 2016 Sep 29;11(9):e0163862. doi: 10.1371/journal.pone.0163862 (PMC5042516; doi:10.1371/journal.pone.0163862)

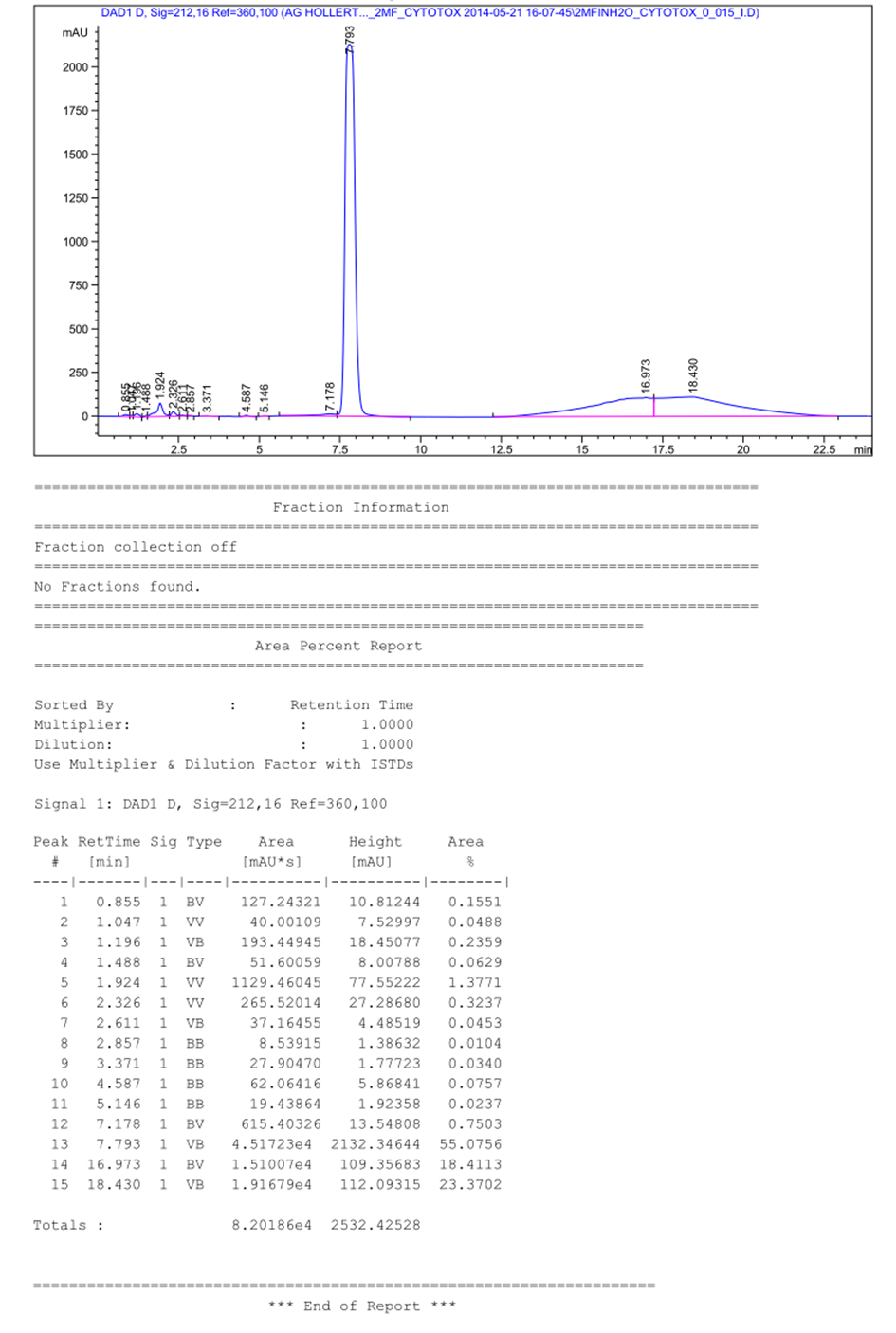

Supplement: S1 File — (TIF) [file pone.0163862.s006.tif]

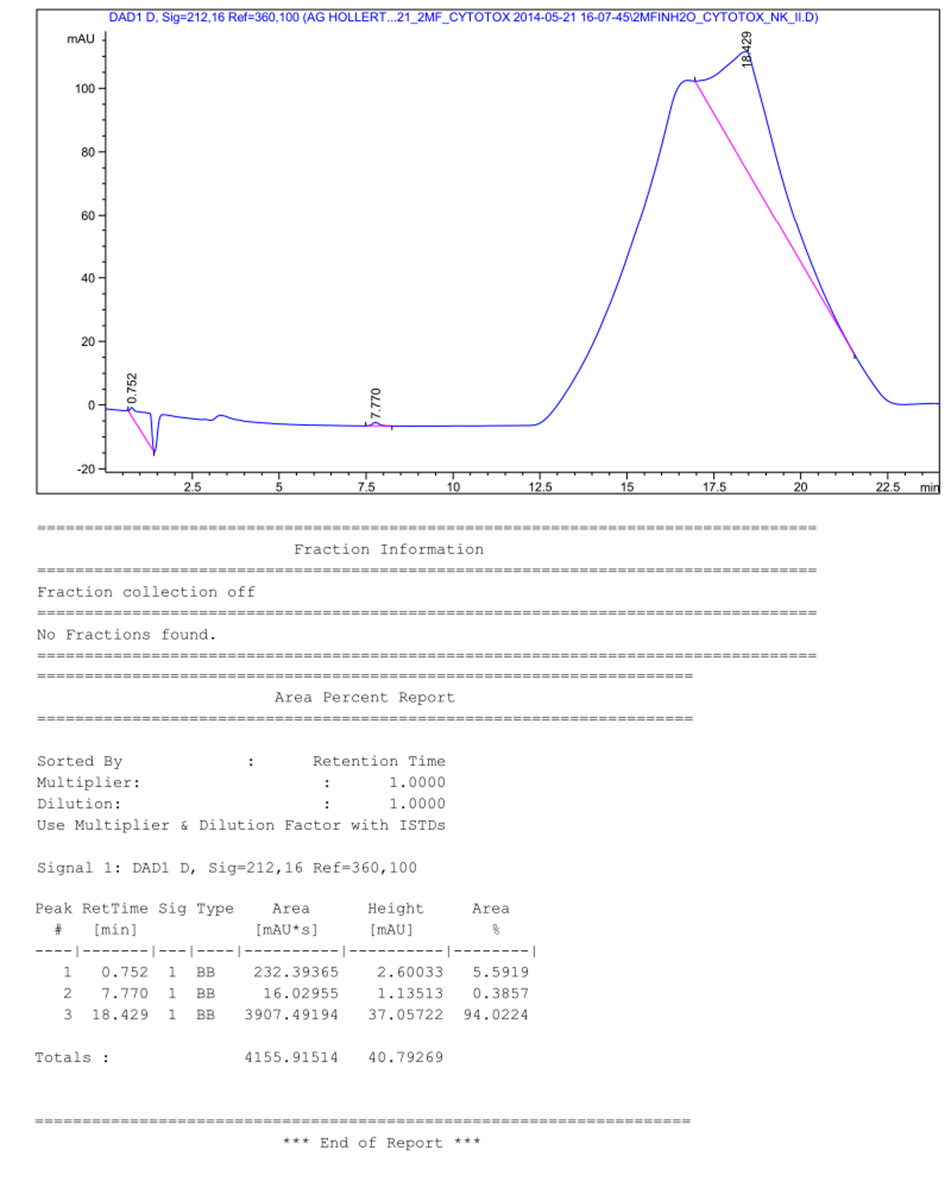

Supplement: S2 File — (TIF) [file pone.0163862.s007.tif]
